# Altered resting-state functional activity in isolated pontine infarction patients with pathological laughing and crying

**DOI:** 10.18632/oncotarget.19307

**Published:** 2017-07-17

**Authors:** Tao Liu, Jianjun Li, Shixiong Huang, Changqinq Li, Zhongyan Zhao, Guoqiang Wen, Feng Chen

**Affiliations:** ^1^ Department of Neurology, Hainan General Hospital, Haikou 570311, China; ^2^ Department of Radiology, Hainan General Hospital, Haikou 570311, China

**Keywords:** pathological laughing and crying, resting-state fMRI, amplitude of low-frequency fluctuation, regional homogeneity

## Abstract

We used resting-state functional magnetic resonance imaging to investigate the global spontaneous neural activity involved in pathological laughing and crying after stroke. Twelve pathological laughing and crying patients with isolated pontine infarction were included, along with 12 age- and gender-matched acute isolated pontine infarction patients without pathological laughing and crying, and 12 age- and gender-matched healthy controls. We examined both the amplitude of low-frequency fluctuation and the regional homogeneity in order to comprehensively evaluate the intrinsic activity in patients with post-stroke pathological laughing and crying. In the post-stroke pathological laughing and crying group, changes in these measures were observed mainly in components of the default mode network (medial prefrontal cortex/anterior cingulate cortex, middle temporal gyrus, inferior temporal gyrus, superior frontal gyrus, middle frontal gyrus and inferior parietal lobule), sensorimotor network (supplementary motor area, precentral gyrus and paracentral lobule), affective network (medial prefrontal cortex/anterior cingulate cortex, parahippocampal gyrus, middle temporal gyrus and inferior temporal gyrus) and cerebellar lobes (cerebellum posterior lobe). We therefore speculate that when disinhibition of the volitional system is lost, increased activation of the emotional system causes pathological laughing and crying.

## INTRODUCTION

Neurological maladies may cause pathological laughing and crying (PLC), a dramatic syndrome characterized by the abnormal expression of emotions [1–3]. The prevalence of PLC in cerebrovascular disease is 11–34%, although the existing data are limited [4]. Patients with this disorder will suddenly begin to laugh or cry, often in episodes that are incongruous with the emotions they are actually feeling [5]. Patients who recount these experiences frequently report that they cry or laugh when they are not sad or do not find anything humorous, and that they cannot cease crying or laughing after the bout begins [6]. A variety of triggers are thought to induce PLC, some of which are mild or indistinct and would not be expected to cause such strong emotions [7]. Patients’ quality of life may be markedly reduced by PLC, due to the humiliating nature of certain outbursts (for instance, the patient may laugh during a funeral). Thus, people with this disorder often limit their social engagement [5].

Although there has been a surge of scientific inquiry about the neural basis of emotional experience and its regulation, little is known about the neural correlates for pathological or normal regulation of emotional expression [8]. A systematic review [8] summarized evidence from animal studies and humans indicating that a volitional system of corticopontine projections from the frontoparietal region (dorsal anterior cingulate gyrus [ACG], posterior insula, primary motor cortex [M1], primary sensory cortex, premotor cortex [PMC], supplementary motor area [SMA] and related parietal regions) suppresses an emotionally–controlled system of frontotemporal projections (anterior insula, inferior temporal gyrus [ITG], orbitofrontal cortex, parahippocampal gyrus and ventral ACG). The latter system normally targets the amygdala–hypothalamus–periaqueductal gray (PAG)–dorsal tegmentum complex controlling the presentation of emotions. Thus, we hypothesized that PLC involves the alteration of multiple frontal-subcortical neuronal circuits, especially the affective network (AN).

The brain regions known as the default mode network (DMN) are most active when individuals are at rest, but are made inactive when individuals perform externally oriented activities [9]. The DMN consists of the medial prefrontal cortex (mPFC), the medial, lateral and inferior parietal cortices, and the precuneus/posterior cingulate cortex. Since the discovery of the DMN, there has been growing interest in its clinical utility and implications [10–13]. The clinical significance of the DMN has been established or implicated in neurological and neuropsychiatric disorders [14–16]. This may be related to potential functions of the DMN, including consolidating the memory [17], improving working memory [18–22], constantly sampling a wide range of external and internal milieu [9, 12], processing stimuli with emotional salience [23], and regulating the interactions of emotional processing and cognitive function [9, 24, 25]. Therefore, we speculate that it may also be abnormal in PLC.

An excellent method for examining spontaneous brain activity is resting-state functional magnetic resonance imaging (rs-fMRI) [26]. Global rs-fMRI signals can be analyzed in terms of the amplitude of low-frequency fluctuation (ALFF) and regional homogeneity (ReHo) [27]. While the neural activity intensity of an individual voxel can be measured by ALFF [28], the neural synchronization between a particular voxel and those that neighbor it can be measured by ReHo [29]. Regional abnormalities may be identified with greater sensitivity by ReHo than by ALFF, while these techniques may complement each other in global spontaneous activity assessment [30]. Thus, to determine the pathophysiological structure of the human brain, it may be better to combine these methods than to use one of them alone [30].

In this study, we used both ALFF and ReHo to examine the global spontaneous brain activity in individuals exhibiting PLC after a stroke. Determining such neural functional alterations would help to elucidate the relevant pathological emotion expression mechanisms responsible for PLC after stroke.

## RESULTS

### Demographics and clinical characteristics

This study included patients admitted to the Neurology Department of Hainan General Hospital between January 2012 and July 2015. We recruited 12 isolated pontine infarction patients with PLC, 12 isolated pontine infarction patients without PLC, and 12 healthy control subjects.

The post-stroke pathological laughing and crying (PSPLC) patients, non-PLC patients and healthy controls did not differ in age or sex. The PSPLC patients’ first PLC episodes occurred in the first month (2/16.67%), second month (5/41.67%), third month (1/8.33%), fourth month (1/8.33%), fifth month (1/8.33%) or sixth month (1/8.33%) post-stroke. Of the PSPLC patients, eight presented with crying only (66.67%), two presented with both crying and laughing (16.67%), and two presented with laughing only (16.67%). When we quantified different features of laughing and crying (the association with external circumstances, the length, the extent of voluntary control, the inconsistency with the patients’ emotions, and the level of resultant distress of these patients), the total scores on the PLC scale were all greater than 13 (18 ± 4) see Table 1. Patients with isolated pontine infarction were recruited to eliminate the effects of multiple lesions on MRI data processing. Notably, bilateral lesions were more common in the PSPLC group than in the non-PLC group (83.33% *vs* 41.67%, *P* = .035) see Table 2. In the PSPLC group, bilateral paramedian basal lesions were observed in four patients, while bilateral paramedian basal–tegmental lesions were observed in six patients.

**Table 1:** Demographics and clinical characteristics of participants

Variables	PLC (*N* = 12)	Non-PLC (*N* = 12)	Healthy control (*N* = 12)	Statistics	*P* value
Age	57.42 ± 5.71	56.59 ± 6.17	57.16 ± 6.24	0.060	0.942
Gender (male/female)	6/6	6/6	6/6	0.000	0.000
Time of the first onset of PLC (months)	1.42 ± 0.51	N/A	N/A		
PLC Scale	18 ± 4	N/A	N/A		

*Note*: unless otherwise indicated, data are means ± standard deviations. PLC, pathological laughing and crying; N/A = not applicable.

The *P* value for gender distribution in the three groups was obtained by a Chi-square test.

The *P* value for age difference between the three groups was obtained by one-way analysis of variance. *P < 0.05.

**Table 2:** Pontine lesion location in PSPLC and control patients

Infarct location	PLC (*N* = 12) *n* (%)	Non-PLC (*N* = 12) *n* (%)	Statistics	*P* value
Bilateral	10 (83.33)	5 (41.67)	4.444	0.035
Paramedian basal lesion	4 (33.33)	4 (33.33)		
Paramedian basal–tegmental lesion	6 (50)	1 (8.33)		
Unilateral	2 (16.67)	7 (58.33)	4.444	0.035
Left/right basal lesion	2 (16.67)	6 (50)		
Left/right tegmental lesion	0 (0)	1 (8.33)		

### ALFF and ReHo results

**Figure 1:**
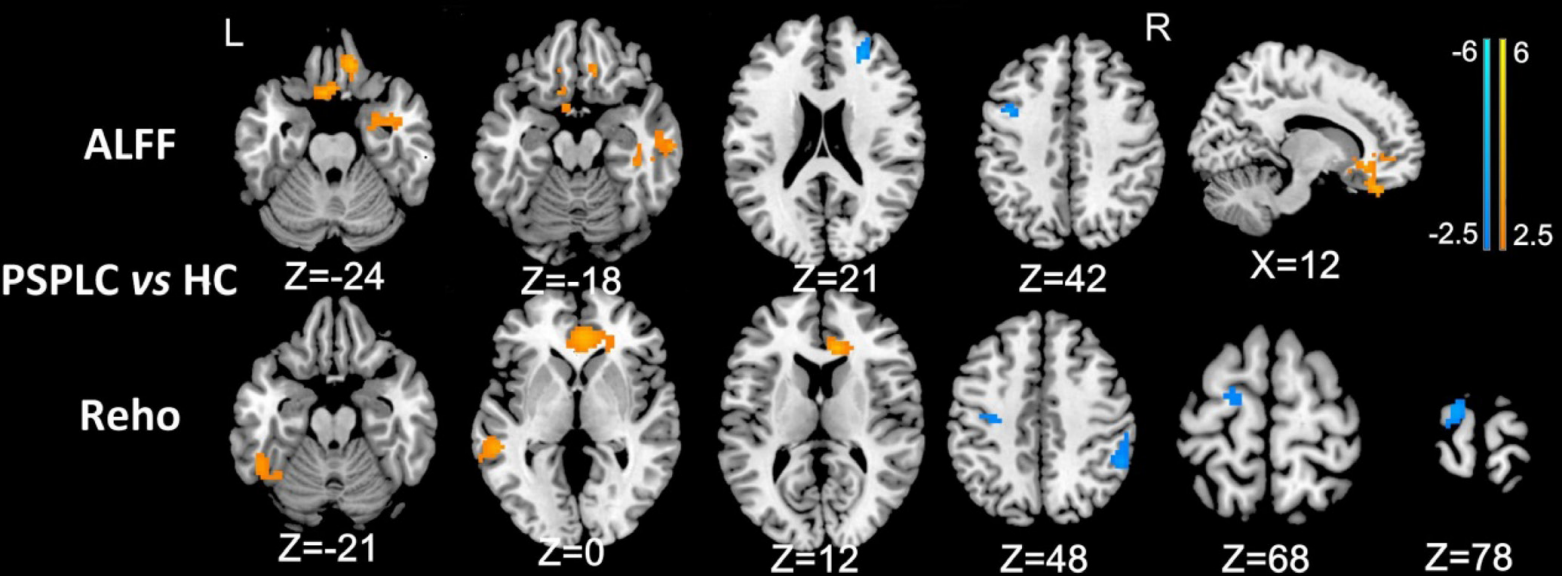
Differences in ALFF and ReHo values between PSPLC patients and healthy controls (*P* < 0.01, AlphaSim corrected) In patients with PLC, the ALFF in the right ACC, MTG, parahippocampal gyrus and bilateral mPFC was significantly greater, and the ALFF in the left precentral gyrus and right SFG was significantly lower than in healthy subjects. In patients with PLC, the ReHo in the left ITG, MTG and bilateral ACC was significantly greater, and the ReHo in the left precentral gyrus, IPL and right SFG/SMA was significantly lower than in healthy subjects.

In patients with PLC, the ALFF in the right anterior cingulate cortex (ACC), middle temporal gyrus (MTG), parahippocampal gyrus and bilateral medial prefrontal cortex (mPFC) was significantly greater, and the ALFF in the left precentral gyrus and right superior frontal gyrus (SFG) was significantly lower than in healthy subjects, as determined by a two-sample *t* test. Additionally, in patients with PLC, the ReHo in the left ITG, MTG and bilateral ACC was significantly greater, and the ReHo in the left precentral gyrus, SFG/SMA and right inferior parietal lobule (IPL) was significantly lower than in healthy subjects (Figure 1; all *P* values < 0.01, AlphaSim corrected).

**Figure 2:**
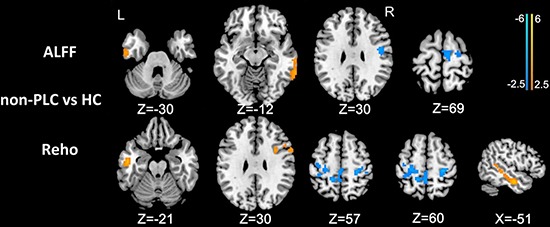
Differences in ALFF and ReHo values between non-PLC patients and healthy controls (*P* < 0.01, AlphaSim corrected) In non-PLC patients, the ALFF in the left ITG and right MTG was significantly greater, and the ALFF in the right precentral gyrus and MFG/SMA was significantly lower than in healthy subjects. In non-PLC patients, the ReHo in the left MTG and right dlPFC was significantly greater, and the ReHo in the left paracentral lobule/precentral gyrus and right precentral gyrus was significantly lower than in healthy subjects.

In non-PLC patients, the ALFF in the left ITG and right MTG was significantly greater, and the ALFF in the right precentral gyrus and middle frontal gyrus (MFG)/SMA was significantly lower than in healthy subjects. Further, in non-PLC patients, the ReHo in the left MTG and right dorsolateral prefrontal cortex (dlPFC) was significantly greater, and the ReHo in the left paracentral lobule/precentral gyrus and right precentral gyrus was significantly lower than in healthy subjects (Figure 2; all *P* values < 0.01, AlphaSim corrected).

**Figure 3:**
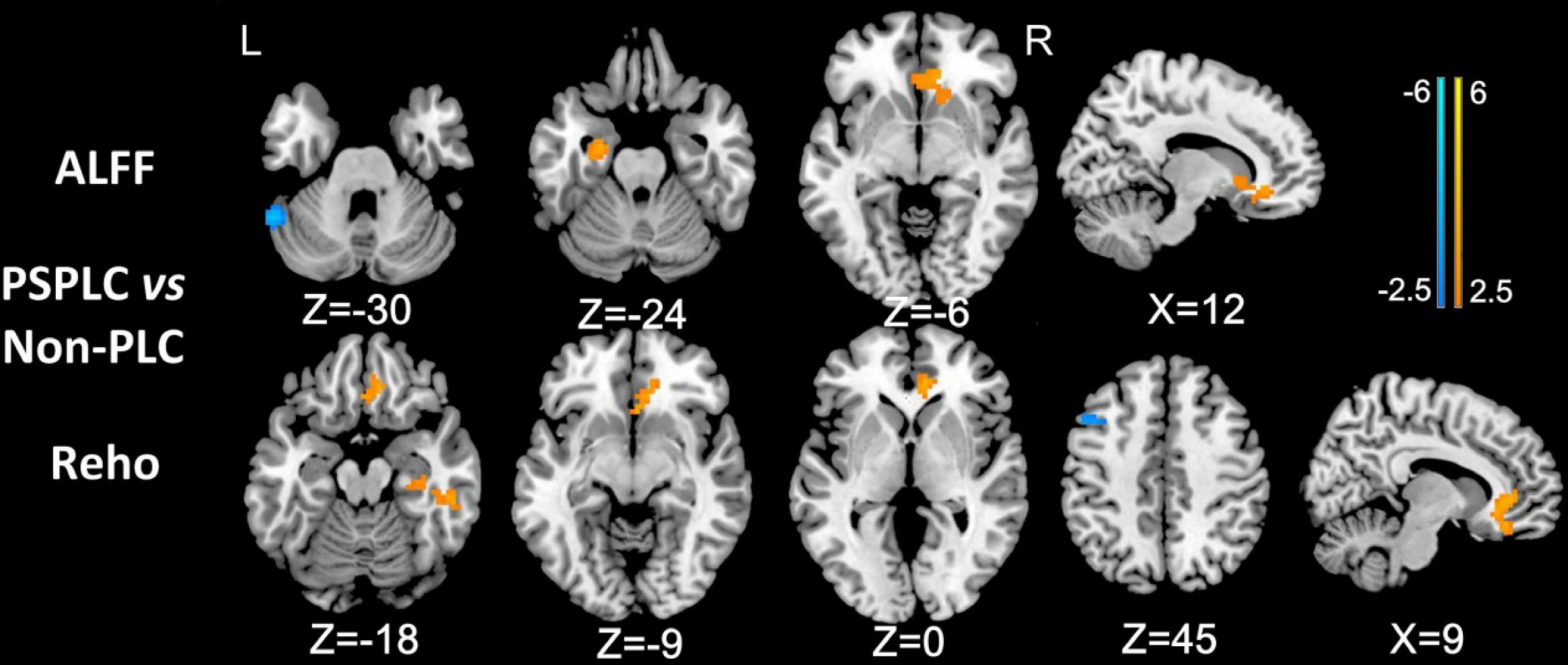
Differences in ALFF and ReHo values between PSPLC patients and non-PLC patients (*P* < 0.01, AlphaSim corrected) In patients with PLC, the ALFF in the right ACC/mPFC and left parahippocampal gyrus was significantly greater, and the ALFF in the left CPL was significantly lower than in non-PLC patients. In patients with PLC, the ReHo in the right ACC/mPFC and ITG was significantly greater, and the ReHo in the left dlPFC was significantly lower than in non-PLC patients.

In patients with PLC, the ALFF in the right ACC/mPFC and parahippocampal gyrus was significantly greater, and the ALFF in the left cerebellum posterior lobe (CPL) was significantly lower than in non-PLC patients. In addition, in patients with PLC, the ReHo in the right ACC/mPFC and ITG was significantly greater, and the ReHo in the left dlPFC was significantly lower than in non-PLC patients (Figure 3; all *P* values < 0.01, AlphaSim corrected). These results are shown in Figures 1–3 and Table 3.

**Table 3:** Differences in ALFF and ReHo values among PSPLC patients, non-PLC patients and healthy controls (P < 0.01, AlphaSim corrected)

	Brain region	BA	Voxels	Maximal t value	MNI coordinates		
					X	Y	Z
PSPLC *vs* HC							
ALFF differences	R.Parahippocampal	21	65	3.54	27	−3	−27
	L/R.mPFC, R.ACC	11,47	178	3.91	12	36	−24
	R.MTG	21	53	3.23	63	−18	−18
	L.Precentral	6	73	−3.58	−42	6	42
	R.SFG	10	65	−3.28	27	45	21
ReHo differences	L.ACC/R.ACC	32,24	312	4.31	12	33	12
	L.ITG	37	42	2.85	−48	−48	−21
	L.MTG	21	69	3.50	−54	−36	0
	L.SFG/SMA	6	74	−3.34	−15	−9	78
	L.Precentral	6	49	−3.08	−36	−15	42
	R. IPL	2,40	118	−3.45	54	−45	45
non-PLC *vs* HC							
ALFF differences	R.MTG	21	80	3.05	66	−45	−12
	L.ITG	20	64	2.89	−54	−12	−30
	R.MFG/SMA	6	72	−2.76	6	−12	69
	R.Precentral	6	73	−3.12	45	−6	30
ReHo differences	R.dlPFC	9	63	2.90	33	9	30
	L.MTG	21	206	4.00	−51	−9	−21
	L.Paracentral Lobule/ L.Precentral	4,6	299	−3.65	−15	−42	57
	R.Precentral	3	59	−3.94	27	−27	60
PSPLC *vs* non-PLC							
ALFF differences	L.Parahippocampal	20	62	3.72	−24	−9	−24
	R.ACC/R.mPFC	32	102	3.92	12	33	−6
	L.CPL		80	−4.61	−54	−51	−30
ReHo differences	R.ACC/R.mPFC	32,25	128	4.03	9	39	0
	R.ITG	20	64	3.54	51	−30	−18
	L.dlPFC	8	45	3.01	−42	30	45

*Note*: Comparisons were performed at *P* < 0.01, corrected for multiple comparisons. Abbreviations: HC: healthy control; L.: left; R.: right; BA: Brodmann's area; *T*: statistical value of peak voxel with significant ALFF and ReHo differences between the two groups (negative values: < Controls; positive values: > Controls); MNI: Montreal Neurological Institute Coordinate System or Template; x, y, z: coordinates of primary peak locations in the MNI space.

## DISCUSSION

As far as we know, this study was the first to use rs-fMRI to investigate the potential alterations of brain function *in vivo* in individuals with PSPLC. Both ALFF and ReHo were examined to comprehensively evaluate the intrinsic activity in patients with PSPLC. Our study revealed changes in these measures in the PSPLC group, mostly in the DMN, sensorimotor network (SMN), AN, and cerebellar lobes. Our findings suggest that there are defects in the resting-state functional architecture in terms of emotional experience and regulation in stroke patients with PLC.

We observed alterations in the ALFF and ReHo at several sites in both the PSPLC and non-PLC groups, including the mPFC/ACC, MTG, ITG, SFG, MFG and IPL, which belong to the DMN. Along with regions of the brain that are made inactive when an individual performs challenging cognitive activities and that inversely correlate with the fronto-parietal areas [12, 31–33], the DMN may be the most important part of the “resting” brain network [9]. The DMN mainly processes information that is relevant to oneself and focused internally [33]. While DMN function is known to be impaired post-stroke [34] (as supported by the present results), such changes also occur in a variety of other disorders, so they are probably not stroke-specific [14, 15]. In stroke patients, changes in the DMN may be associated with conditions such as discomfort and chronic pain [35]. These subjective emotions may be exacerbated in PLC, corresponding to the greater disturbances in PLC than in non-PLC patients. On the other hand, the DMN processes stimuli of emotional salience [23] and regulates the interactions of emotional processing and cognitive function [9, 24, 25], further explaining the abnormal brain activity in PSPLC group. However, further research is needed to assess neurocognition with unbiased and specific methods.

Our analyses of ALFF and ReHo indicated that integration was impaired in the SMN (SMA, precentral gyrus and paracentral lobule) of resting-state PSPLC and non-PLC patients. The SMN [36], which is important for voluntary motion, links primary motor cortex (M1) function with SMA function [37, 38]. By abruptly disturbing brain activity, strokes impede neural networks needed for voluntary motion. Studies of animal models of stroke have revealed a complex cascade of events that alter structural connections and synaptic transmission in brain regions that are both proximal and distal to the lesion [39]. For example, tract-tracing studies in rats have demonstrated that experimental lesions of the primary sensory cortex (S1) are followed by axonal sprouting from cell bodies adjacent to the lesion into the peri-infarct cortex [40]. Likewise, new projections arise from S1 to the ventral PMC after lesions to M1 [41]. Considering these prior results and those of the present study, we contend that strokes can disrupt networks close to the lesion as well as in distant regions of the cortex in either hemisphere.

In the PSPLC group, we also observed increases in spontaneous brain activity in ALFF and ReHo analyses of the AN (mPFC/ACC, parahippocampal gyrus, MTG and ITG). Numerous human studies have indicated that happiness and sadness are perceived, experienced, recalled and expressed through an emotional network that includes the ACG, amygdala, anterior temporal pole, anterior and posterior temporal lobes, basal temporal cortex, hippocampus, left ventrolateral prefrontal cortex, mPFC, OBF and parahippocampal gyrus [42–49]. The anterior insula, cingulate gyrus (CG), mesial temporal cortex, superior temporal gyrus and transverse temporal gyrus have also been implicated in sadness, whereas the dlPFC, ITG, mesial frontal cortex and occipitotemporal (fusiform) gyrus have been further implicated in happiness [8]. At least to some degree, the increased spontaneous brain activity in the AN may correlate with PLC and indicate that emotional management and behavioral function are disrupted in PSPLC.

The AN areas also belong to an emotionally controlled system regulating emotional expression [8]. Haiman et al. [50] evaluated multiple sclerosis (MS) patients with and without involuntary emotional expression disorder (IEED) and healthy controls matched for age and sex (11 subjects per group) for their evoked potentials. Following emotional stimuli of subjective significance, the current density in several areas (including the M1 and SMA) differed according to IEED status [50]. In another report [51], these authors evaluated six MS patients with IEED before and after dextromethorphan–quinidine (DM-Q) treatment and six untreated healthy controls for their evoked response current density. The treatment normalized the evoked potentials, diminished the current density in the ACG, ITG, MFG, M1, supramarginal gyrus and right uncus, and elevated the current density in the CG, middle occipital gyrus and inferior occipital gyrus when stimuli of subjective significance were provided [51]. The system responsible for the volitional expression of emotions is predominantly operated by the dorsal cingulate gyrus, M1, PMC, posterior granular and dysgranular insula, pre-SMA, SMA, S1 and related parietal cortices. Considering these previous findings and the present results, we speculate that when the disinhibition of the volitional system is lost, increased activation of the emotional system causes PLC.

Interestingly, we found that the ALFF in the left CPL was lower in the PSPLC group than in the non-PLC group. Given the evoked potentials and the responses to DM-Q in MS patients with IEED, along with the similar structures that are associated with PLC and pain, Haiman and colleagues [50] proposed using gate theory to explain PLC, just as it is used to explain pain. The authors posited that lower activity of volitional/voluntary pathways due to lesions, greater activity of emotional/involuntary pathways in response to stimuli, or lower activity of PAG or raphe magnus pathways may cause PLC [50]. Miller and colleagues [52] further proposed that such gating is made possible by appropriate synaptic connections in the cerebellum, and that cerebello-cortical projections subsequently affect thresholds for the expression of emotions in the sensorimotor cortex, even if comparable cerebellar pain models [53] depend on gating of the brain stem [54] and PAG [55]. Further research is needed to validate this conclusion.

This study had the following limitations. Firstly, we selected patients with PLC limited to isolated pontine infarction, which cannot represent PLC associated with other stroke locations. Since strokes have heterogeneous impacts on structure and function, we would have gained further insight by recruiting patients with lesions in different areas, instead of choosing only those with pontine infarction. Secondly, the sample size was relatively small.

In conclusion, in PSPLC, the prominent changes in neural activity were observed in the DMN (mPFC/ACC, MTG, ITG, SFG, MFG and IPL), SMN (SMA, precentral gyrus and paracentral lobule), AN (mPFC/ACC, parahippocampal gyrus, MTG and ITG) and cerebellum, which may reflect the neural plasticity of the cerebellar functional network in PLC.

## MATERIALS AND METHODS

### Ethics statement

This study was approved by the research ethics review board of the Hainan General Hospital, Haikou, China, according to the Declaration of Helsinki (2000). Each subject read and signed the consent form before being included in the study.

### Inclusion and exclusion criteria

The following were the diagnostic criteria for PSPLC [56]: (1) confirmed diagnosis of isolated pontine infarction by MRI; (2) recurrent outbursts of uncontrolled crying or laughing without any concomitant change in mood in the six months after stroke, with symptoms lasting for at least two weeks; (3) a total score of 13 or higher on the PLC scale, which is both valid and reliable in quantitatively assessing the severity of PLC symptoms in stroke patients [57]; and (4) uncontrolled crying or laughing that could not be explained by other neurological or psychological diseases. The interview of the patient was conducted in the presence of a close relative. If no relative was present during the interview, a telephone call was made to confirm the patient's answers. The exclusion criteria were (1) dacrystic or gelastic seizures; (2) cramps or dystonia of facial muscles; (3) a history of amyotrophic lateral sclerosis, multiple sclerosis, Alzheimer's disease, Parkinson's disease, traumatic brain injury or intracranial tumor; (4) depression or other psychiatric diseases; and (5) communication problems (disturbance of consciousness or aphasia) severe enough that the patient could not complete a reliable interview.

Acute isolated pontine infarction patients who did not demonstrate PLC in clinical evaluation were enrolled as a comparison group. During the initial consultation, the following information was obtained from each patient: sex, age at which the stroke occurred, time elapsed since the stroke, and location and laterality of the lesion. An age- and gender-matched group of healthy volunteers served as the control group. All control subjects had normal neurological examinations, no stroke history and no significant active neurological problems. They were also free of systemic diseases and neurological disorders. All subjects were strongly right-handed according to the Edinburgh Handedness Inventory.

### MRI data acquisition

MRI data were obtained on a Siemens Verio3T MRI scanner with a standard six-channel head coil (Erlangen, Germany) in the Department of Radiology, Hainan General Hospital. Subjects were instructed to keep their eyes closed but to remain awake, to avoid thinking of anything in particular, and to keep their heads still during the scanning. If the patient had an episode of PLC during the MRI scan, the procedure was terminated until the episode subsided. A routine-structure MRI scan was conducted to exclude gross cerebral pathology. Anatomical images of the functional slice locations were obtained with spin-echo imaging in the axial plane parallel to the Anterior Commissure-Posterior Commissure line. Whole-brain functional images were collected by a T2*-weighted EPI sequence sensitive to BOLD contrast (repetition time = 2000 ms, echo time = 30 ms, field of view = 240 × 240 mm^2^, flip angle = 80°, image matrix = 64 × 64, no gap, voxel size = 3.75 × 3.75 × 5 mm^3^; each brain volume comprised 31 axial slices and each functional run contained 240 volumes). A high-resolution T1-weighted structural image was acquired with a magnetization-prepared rapid gradient-echo (MPRAGE) sequence (repetition time = 2300 ms, echo time = 2.9 ms, TI = 900 ms, field of view = 256 × 256 mm, flip angle = 9°, in-plane matrix = 256 × 256, slice thickness = 1 mm, no gap, and voxel dimensions = 1 × 1× 1.33 mm^3^).

### MRI data post-processing

Pre-processing of fMRI data was performed with the toolbox Data Processing Assistant for Resting-State functional MR imaging (DPARSF; http://www.restfmri.net/forum/DPARSF) through Statistical Parametric Mapping (SPM8; http://www.fil.ion.ucl.ac.uk/spm/) and an rs-fMRI data analysis toolkit (REST1.8; http://www.restfmri.net). The first 10 volumes of each functional time series were discarded for the magnetization equilibrium. Slice timing and realignment for head motion correction were performed. Spatial normalization to the standard Montreal Neurological Institute (MNI) echo-planar imaging template in the Statistical Parametric Mapping package. The functional images were then spatially normalized to standard coordinates and resampled to 3 × 3 × 3 mm^3^. Subjects with head motion > 1.5 mm translation or > 1.5° rotation in any direction were excluded.

### ALFF and ReHo analyses

ALFF and ReHo were analyzed with REST software. For ALFF analysis, the resampled images were first smoothed with a Gaussian kernel of 4 mm. Then, linear trend and band-pass filtering (0.01–0.08 Hz) were performed to remove the effects of low-frequency drift and high-frequency noise. Next, the time series was transformed to the frequency domain with a fast-Fourier transform. The raw ALFF value was calculated as the average of the square root of the power spectrum across 0.01–0.08 Hz within each voxel. Afterwards, the raw value of each voxel across the brain was extracted and averaged to calculate the global mean ALFF value. Lastly, the raw ALFF values of all voxels were divided by the global mean ALFF values for standardization. The degree of the raw ALFF value relative to the average ALFF value of the entire brain was reflected in the resulting ALFF value in a specific voxel.

ReHo was also analyzed on preprocessed images. We obtained ReHo images by calculating the concordance of the Kendall coefficient of the time series of a specific voxel with its nearest neighbors after linear trend and band-pass filtering were carried out. We then standardized the ReHo value of each voxel by dividing the raw value by the global mean value, which was acquired by the same calculation when the global mean ALFF value was determined. Lastly, we smoothed the data with a Gaussian kernel of 4 mm for further statistical analysis.

### Statistical analysis

Demographic and clinical variables were compared among the three groups with SPSS software (version 16.0; SPSS, Inc., Chicago, IL, USA). Analysis of variance was used for continuous variables, and a Chi-square test was performed for proportions. *P* values < 0.05 were considered statistically significant.

The between-group differences in ALFF and ReHo values were determined by two-sample *t* tests performed with the REST software (within a brain mask). The statistical threshold was set at *P* < 0.01 and a minimum cluster size of 20 voxels, which corresponded to a corrected *P* < 0.01 (AlphaSim correction).

## Abbreviations

ACC: Anterior cingulate cortex
ACG: anterior cingulate gyrus
ALFF: amplitude of low-frequency fluctuation
AN: affective network
CPL: cerebellum posterior lobe
dlPFC: dorsolateral prefrontal cortex
DMN: default mode network
DM-Q: dextromethorphan–quinidine
IEED: involuntary emotional expression disorder
IPL: inferior parietal lobule
ITG: inferior temporal gyrus
M1: primary motor cortex
MFG: middle frontal gyrus
MPFC: medial prefrontal cortex
MS: multiple sclerosis
MTG: middle temporal gyrus
PAG: periaqueductal gray
PLC: Pathological laughing and crying
PMC: premotor cortex
PSPLC: post-stroke pathological laughing and crying
ReHo: regional homogeneity
rs-fMRI: Resting-state functional MR imaging
SFG: superior frontal gyrus
SMA: supplementary motor area
SMN: sensorimotor network
